# Double Up Food Bucks program effects on SNAP recipients' fruit and vegetable purchases

**DOI:** 10.1186/s12889-017-4942-z

**Published:** 2017-12-12

**Authors:** Marie Steele-Adjognon, Dave Weatherspoon

**Affiliations:** Agriculitural, Food and Resource Economics Department, Michigan State University, 466 W Circle Drive, East Lansing, MI 48824 USA

**Keywords:** Fruits and vegetables, Program evaluation, Detroit, Scanner data, Supplemental nutrition assistance program

## Abstract

**Background:**

To encourage the consumption of more fresh fruits and vegetables, the 2014 United Sates Farm Bill allocated funds to the Double Up Food Bucks Program. This program provided Supplemental Nutrition Assistance Program beneficiaries who spent $10 on fresh fruits and vegetables, in one transaction, with a $10 gift card exclusively for Michigan grown fresh fruits and vegetables. This study analyzes how fruit and vegetable expenditures, expenditure shares, variety and purchase decisions were affected by the initiation and conclusion, as well as any persistent effects of the program.

**Methods:**

Changes in fruit and vegetable purchase behaviors due to Double Up Food Bucks in a supermarket serving a low-income, predominantly Hispanic community in Detroit, Michigan were evaluated using a difference in difference fixed effects estimation strategy.

**Results:**

We find that the Double Up Food Bucks program increased vegetable expenditures, fruit and vegetable expenditure shares, and variety of fruits and vegetables purchased but the effects were modest and not sustainable without the financial incentive. Fruit expenditures and the fruit and vegetable purchase decision were unaffected by the program.

**Conclusions:**

This study provides valuable insight on how a nutrition program influences a low-income, urban, Hispanic community’s fruit and vegetable purchase behavior. Policy recommendations include either removing or lowering the purchase hurdle for incentive eligibility and dropping the Michigan grown requirement to better align with the customers’ preferences for fresh fruits and vegetables.

**Electronic supplementary material:**

The online version of this article (10.1186/s12889-017-4942-z) contains supplementary material, which is available to authorized users.

## Background

There is extensive evidence of the health benefits associated with eating fruits and vegetables (F&V); however, many Americans consume significantly less than the recommended daily level of F&V according to federal guidelines [1, 2]. In an attempt to help address these dietary deficiencies, the 2014 Farm Bill allocated $100 million over 5 years for the Food Insecurity Nutrition Incentive (FINI) [3, 4]. FINI is a grant program designed specifically to support programs aimed at increasing F&V consumption among Supplemental Nutrition Assistance Program (SNAP)^1^ participants [5]. One of the programs selected to receive funding from FINI was the Double Up Food Bucks (DUFB) program.

The DUFB program provides SNAP customers that spend $10 on fresh F&V (in one transaction) with a $10 gift card exclusively for Michigan grown fresh F&V. The DUFB gift card was activated by the store cashier immediately after the completion of the $10 F&V purchase transaction and was valid until the end of the program. DUFB is unique in that it provides a financial incentive for Michigan grown F&V only, while most other programs do not have a locally grown F&V restriction [1, 6]. DUFB is also unique with respect to its relatively large purchase hurdle followed by a lump-sum financial transfer.

The pilot project for DUFB was conducted in Detroit, Michigan; partially because it has a large proportion of its residents living in a food desert, the largest depopulation rate from 2000 to 2010, and in 2013 the city filed for bankruptcy and has yet to fully recover [7]. Low income urban communities with high poverty rates, like this study site, typically have high obesity rates and substantial dietary deficiencies (including insufficient F&V consumption) [8, 9]. DUFB has expanded to 17 states [10]; hence, determining if DUFB can increase the purchase and possibly the consumption of fresh F&V by SNAP recipients is critical to food policy.

Supermarket based programs aimed at increasing F&V consumption have been implemented within supermarkets over time. These interventions typically fall into one (or more) of four classifications: (1) point-of-purchase information, (2) increased availability, variety, and convenience, (3) promotion and advertising, and (4) financial incentives [11]. Studies have found mixed results as to whether supermarket based programs are effective at increasing consumers’ nutritional knowledge and consumption of F&V and most of those that did find influential effects, found the effects were not sustainable over time [12–15].

The purpose of this paper is to evaluate the effects of the DUFB program on fresh F&V purchases in a low-income community in Detroit. Specifically, how did DUFB implementation impact F&V purchase behaviors, how the conclusion of DUFB impacted purchase behaviors and did DUFB have persistent effects. This evaluation is unique in that it utilizes scanner data from a supermarket to evaluate a nutrition program versus interviews [16], surveys [17], receipt collecting [18], and 24-h food recalls [19]. Hence, the data does not contain self-report response bias but reflects what is purchased, which may not represent what was consumed. Another unique aspect of this analysis is that fixed effects estimation was used to control for unobserved heterogeneity, which provides a more reliable estimate of the program’s impact. Lastly, most studies conducted in the U.S. do not examine Hispanic neighborhoods, even though the literature shows that food demand differences exist among ethnic groups in America [20, 21].

## Methods

### Data

Scanner data from a Detroit independent supermarket that participated in DUFB was used for this study. The store is located in a low-income predominantly Hispanic community (within the census tract Hispanic is the primary ethnicity; 69% of households are families; 90% have not attended any type of college and the median household annual income is under $30,000 [22]). The data includes all store transactions from May 2014 through January 2015. The unformatted receipt text file was converted into a Stata file using Python version 2.7.2. A unique identifier was created for 41% of the transactions where the customer either had a loyalty card, credit card, debit card, SNAP benefits card or a WIC account. The data was then transformed into a panel dataset where each observation represents a customer’s monthly purchases. The panel dataset structure allows the comparison of F&V expenditures over time for each consumer with a unique identifier.^2^

### Non-random treatment assignment

This study estimates the causal effects of the DUFB program using a quasi-experimental approach. Eligibility of customers to participate was nonrandom given that only SNAP beneficiaries were eligible to participate in DUFB. Difference in Difference (DD) relies on data of both the treated and the control groups before and after treatment, to control for any confounding effects present, in order to estimate the treatment effects [23]. This panel dataset permits the use of both a cross-sectional estimator and a time-series estimator to difference away any permanent differences between the groups and any common trends affecting both groups; hence, the non-random treatment assignment of DUFB was addressed by using DD (see Additional file 1 for assessment of the DD parallel trend assumption).

The DD approach is represented by:

$$ \mathrm{DD}=\mathrm{E}\left({\mathrm{Y}}_1^{\mathrm{T}}-{\mathrm{Y}}_0^{\mathrm{T}}\ \right|\ {\mathrm{T}}_1=1\left)-\kern0.5em \mathrm{E}\left({\mathrm{Y}}_1^{\mathrm{C}}-{\mathrm{Y}}_0^{\mathrm{C}}\ \right|\ {\mathrm{T}}_1=0\right) $$

where T_1_ = 1 denotes that the customer is treated and T_1_ = 0 otherwise. ${Y}_t^T$ and ${Y}_t^C$ are the F&V purchase behavior for the treated and non-treated customers, respectively, in the initial time period (*t* = 0) and the final time period (*t* = 1).

### F&V purchase behaviors

There are six different F&V purchase behaviors that were examined to determine the effects of DUFB: F&V expenditure; fruit expenditure; vegetable expenditure; F&V expenditure share; F&V variety; and F&V purchase decision. F&V expenditure is the aggregate dollar amount spent during the month on all fresh F&V. Fruit expenditure and vegetable expenditure are the independent allocation of those expenditures, which reveals how each are individually affected. F&V expenditure shares measure the ratio of fresh F&V purchases to all other store purchases to identify how the F&V expenditures change relative to expenditures in the rest of the store. Variety of F&V is a count of the different F&V purchased during the month, which captures whether the program increased the diversity of F&V purchased. The F&V purchase decision is the customer’s binary decision to purchase F&V or not, to illustrate whether the program incentivized customers to purchase F&V. Evaluating these purchase behaviors reveals the potential effects of the program.

### Program time indicator variables

The dataset was divided into three time periods: before DUFB (May 1, 2014 – July 31, 2014), during DUFB (Aug 1, 2014 – Nov 30, 2014), and after DUFB (Dec 1, 2014 – Jan 31, 2015). The three time periods allow the analysis of the following purchase behavior comparisons: before versus during DUFB to determine how the implementation impacted purchase behaviors; during versus after DUFB to determine how the conclusion of DUFB impacted purchase behaviors; and before versus after to determine if DUFB had any persistent effects. To examine the initial DUFB incentive effect, the time variable, T, was defined as 0 if the observation was before DUFB and 1 if during DUFB. To measure whether the conclusion of DUFB has an effect, the time variable, T, was redefined as 0 if the observation was during the DUFB implementation and 1 if after the implementation. Whether DUFB has a persistent effect or not was measured through redefining the time variable, T, to 0 if the observation was before the DUFB implementation and 1 if after the implementation.

## Results

Table 1 provides descriptive statistics of the shopping behaviors of 12,699 unique identifiable customers (those customers with ID numbers). The average customer for this store spent $83.69 overall and $4.92 on F&V per month. For those customers that purchased F&V at least once per month, their average expenditure was $8.60. The average F&V expenditure share was 6.21%, a little more than half the national average F&V expenditure share from supermarkets (11.6%) [24]. The average customer spent more on vegetables than fruits.

**Table 1:** Descriptive Statistics of the Panel Dataset

	Monthly Mean over the Entire Dataset	SNAP Customers Monthly Mean Before DUFB
Store Expenditure	$83.69	$98.98
F&V Expenditure	$4.92	$5.21
Conditional F&V Exp ^a^	$8.60	$8.12
F&V Expenditure Share	6.21%	5.63%
Fruit Expenditure	$2.11	$2.42
Vegetable Expenditure	$2.82	$2.86
F&V Variety	2.19	2.47
Number of Visits	2.67	2.79
	Number of Unique Customers	Percent of All Unique Customers
With ID	12,699	100
Purchased F&V at least once	10,152	79.9
Purchased F&V each month	2301	18.1
Paid with SNAP ^b^	7880	62.1
Paid with Credit or Debit ^b^	839	6.6
Paid with Cash ^b^	5160	40.6
Paid with WIC ^b^	211	1.7
Loyalty Card Members	3564	28.1

^a^Conditional on F&V being purchased

^b^Payment Methods were not mutually exclusive because if the customer uses a loyalty card they could pay with more than one method within the month and still have the same ID number

Comparing SNAP customers with the rest of the customers at this store shows that they spent more overall and more on F&V, but had lower F&V expenditure shares, on average. SNAP customers who purchased F&V before DUFB, purchased on average $8.12 worth of F&V a month, which is less than the transaction level purchase hurdle that DUFB requires ($10). This is an initial indicator that the purchase hurdle may be too high to incentivize F&V purchases.

Approximately 80% of the customers purchased a fruit or vegetable at least once in the nine-month period and 18% purchased a fruit or vegetable every month in the nine-month period. On average, customers purchased 2.19 different types of F&V per month. The average shopping frequency at this store was 2.7 times per month, which is low compared to the national average of 6 supermarket visits per month [24]. The number of identifiable customers that shopped during the individual months was relatively steady throughout the 9 months, ranging from 6051 to 6332 customers per month (not shown in Table 1). Approximately 62% of the identifiable customers paid with SNAP benefits during the nine-month period.

### DUFB low participation

DUFB program participation was defined by whether the SNAP customer earned and used the DUFB gift card. The DUFB gift card usage was low at this store, only 1.87% of all SNAP transactions during implementation used a DUFB card to purchase Michigan grown F&V. There were 156 unique customers who used the DUFB card once, 23 used it twice, seven used it three times, and four who used it four or more times during the four-month implementation.^3^ The number of times a customer could spend $10 on F&V and receive $10 for Michigan grown F&V was unlimited during the 4-month implementation period; however, eight was the maximum number of times that a single customer used the program. DUFB participation was low enough that concerns of noncompliance were present; hence, the Intention to Treat (ITT) was estimated [25]. ITT interpretation of results were not biased from this noncompliance of the participants since it was based on the initial treatment assignment rather than on whether or not the customer actually participated [25]. This categorizes all SNAP participants as being treated by DUFB, even though many did not actually receive or redeem their $10 gift card (the effect of being assigned to treatment rather than the effect of receiving treatment); hence the ITT analysis provides a conservative estimate of the treatment effect [26].

### DUFB program effects

All six dependent variables (F&V expenditure, fruit expenditure, vegetable expenditure, F&V expenditure share, F&V variety and the F&V purchase decision) were run for the three program effects based on time, for a total of 18 regressions. Table 2 offers a summary of the DUFB effects on the six F&V purchase behaviors over time. (Additional files 2, 3 and 4 show all the regression results for the initial incentive effects, after incentive effects, and the persistence of program effects.) The linear regression with fixed effects estimates a linear approximation of program effects. This allows the unobserved heterogeneity to be controlled through fixed effects, while offering a clear interpretation of the results across the different program effects [27]. A robustness check for each regression was estimated with their appropriate nonlinear model. (Please see Additional file 5 for nonlinear regressions results.) The linear and non-linear models have similar results in terms of signs and significance but have slightly different magnitudes due to the estimation procedures; only the linear models will be discussed.

**Table 2:** Summary of DUFB Effects over the Three Time Periods

	Before versus During	During versus After	Before versus After
	Average Change (95% Confidence Interval)	Average Change (95% Confidence Interval)	Average Change (95% Confidence Interval)
F&V Expenditure	$0.40*** ($0.12, $0.68)	-$0.27* (−$0.58, $0.05)	$0.07 (−$0.31, $0.44)
Fruit Expenditure	$0.08 (−$0.08, $0.24)	-$0.08 (−$0.25, $0.10)	$0.06 (−$0.16, $0.27)
Veg Expenditure	$0.33*** ($0.14, $0.51)	-$0.19* (−$0.41, $0.02)	$0.01 (−$0.24, $0.26)
F&V Exp Share	0.70%*** (0.27%, 1.12%)	−0.50%** (−0.99%, −0.06%)	−0.10% (−0.64%, 0.45%)
F&V Variety	0.11** (0.010, 0.203)	−0.16*** (−0.27, −0.05)	−0.08 (−0.21, 0.05)
F&V Purchase Decision	0.01 (−0.01, 0.02)	−0.01 (−0.03, 0.01)	−0.02 (−0.04, 0.01)

***p < 0.01

**p < 0.05

*p < 0.1

An increase of $0.40 in the SNAP customers’ monthly F&V expenditures was attributable to the DUFB program being implemented. This implies that over the 4 months that DUFB was implemented, SNAP customers spent a total of $1.60 more on F&V compared to what they would have spent had the DUFB program not been implemented. However, most of that significant increase in expenditure was from increased expenditures on vegetables, which increased by $0.33 a month due to DUFB, while the fruits expenditure did not significantly increase due to DUFB. DUFB increased the F&V expenditure share by 0.7% and the number of F&V varieties purchased by 0.11. DUFB did not influence on the decision to purchase F&V.

The loss of the DUFB financial incentive was responsible for a $0.27 decline in the monthly F&V expenditure by the SNAP participants. Examining the F&V expenditure effects separately reveals that the program decreased monthly vegetable expenditures by $0.19, but had no significant impact on fruit expenditures. These SNAP consumers spent less money on fruits than vegetables, on average, and their fruit consumption was not affected by the implementation nor conclusion of the DUFB program while their vegetable consumption was statistically significantly affected by both. The F&V expenditure shares and the variety of F&V significantly decreased after the DUFB ended by 0.5% and 0.16 F&V, respectively. Similar to the fruit expenditure, the F&V purchase decision was unaffected by the start and the end of DUFB.

The DUFB program did have positive effects on the F&V purchase behaviors and the conclusion of DUFB had negative effects; however, this raises the question as to whether the program had any lasting impact on the purchasing habits of customers. This was investigated by comparing the F&V expenditures before and after DUFB implementation. There were no statistically significant differences in fruit or vegetable expenditures, F&V expenditure shares, F&V variety and the probability of purchasing F&V before or after DUFB was implemented. Hence, DUFB did not have a lasting effect on any of these dimensions of consumer F&V purchasing behavior at this store and all F&V purchasing behaviors returned to where they were before DUFB was implemented.

Other factors that influenced the F&V purchasing behaviors were seasonality, frequency of store visits, and non-F&V store expenditures. Most of the month dummy variables were significant, meaning that seasonality affects customers’ F&V expenditure shares, variety of F&V purchased and the decision to purchase. Fruit expenditure was higher in the warmer months of the year, similar to national data [28]. The number of store visits the consumer makes in a month and the F&V purchasing behaviors were significant and positive for all the program times. The more frequently a customer shops at the store the greater their F&V expenditures, the higher their F&V expenditure share, the more varieties of F&V were purchased and the more likely they were to purchase F&V. Consumers who spend more money throughout the rest of the store, spend more money on both F&V, purchase more varieties of F&V and were more likely to purchase F&V. However, as the non-F&V expenditure level increases, the F&V expenditure share decreases. These relationships were consistent across the program time comparisons.

## Discussion and conclusions

In order to incentivize SNAP participants to consume more F&V, the DUFB program gave $10 gift cards for Michigan grown F&V to SNAP customers that spent $10 on F&V. DUFB did increase SNAP customers’ vegetable expenditures, their F&V expenditure shares and the variety of F&V purchased during implementation; however, persistent program effects on purchasing behaviors were lacking and may require longer interventions, as shown in other studies [29]. We found that the DUFB effects were relatively modest compared to what other financial incentive programs in supermarkets have generated [1, 30, 31]. The Healthy Incentives Pilot program, a subsidy intervention, gave SNAP customers 30% off on targeted F&V purchases and increased F&V expenditures by 20% [1], which was larger than the 5.8% F&V expenditure increase found for DUFB. An intervention implemented in Pennsylvania which gave low-income customers a 50% rebate on fresh and frozen F&V (for 8 weeks), and then a 25% rebate during a tapering phase (for 4 weeks) before ending was also found to be more impactful. Similar to DUFB, this program significantly increased the treated households’ weekly F&V purchases, vegetables more so than fruits, and when the incentive was discontinued, households returned to their baseline F&V purchases [32].

### Program implications

The lack of participation and persistence of program effects are concerning for the DUFB program. DUFB participation was extremely low, evident by only 535 DUFB transactions out of the 28,609 total potential SNAP transactions during DUFB implementation at this store. This low participation rate, especially compared to subsidy type financial incentive programs, could be an indication that the $10 F&V purchase hurdle discouraged participation rather than encouraged spending more. This purchase hurdle requirement prior to any benefits being received in the DUFB program mirrors the early purchase requirements of the U.S. food stamps program which required low-income households to meet food purchase requirements in order to receive food stamps [33]. The large hurdle failed for DUFB as it did for SNAP in the past; hence, one suggestion to increase the participation rate is to make this purchase hurdle smaller.

Prell & Smallwood [34] use neoclassical economics to show that the effectiveness of a program depends on the proportion of consumers who fall into the different consumer spending types. In this community, approximately 70% of the consumers purchase little to no F&V (less than $5 worth a month), 15% purchase a moderate amount (between $5 and $10 a month), and 15% purchase more than $10 worth a month. Consumers who initially purchase no F&V’s are less responsive to initiatives that require them to pay something to participate [34]. This may reveal that a more economically efficient type of incentive program for this community would be a subsidy type program, for example giving a discount on all Michigan produce purchased. Another potential program option is for every dollar a customer spends on F&V be matched in their next transaction. Our results indicate that the program had no significant effects on the consumers’ F&V purchase decision so the store should cross-merchandize and have signage and displays more throughout the rest of the store (outside of produce department) to target those customers who may not typically enter the produce department.

Consumer preferences are important factors to consider in evaluating the effectiveness of consumer behavior based programs. Analyzing the most frequently purchased F&V at this store reveals that the grown in Michigan requirement should be dropped in order to encourage increased F&V consumption. Michigan grown F&V are apples, asparagus, blackberries, blueberries, cabbage, carrots, celery, cherries, corn, cucumbers, onions, peaches, pears, plums, raspberries, strawberries, and tomatoes [35]. Only two of these (apples and peaches) make the list of the top ten sold fruits and five of these (cabbage, carrots, cucumbers, onions and tomatoes) make the list of the top ten sold vegetables at this store. This divergence between preferences and gift card eligibility could be driving the low program participation and the lack of DUFB effect on fruit expenditures.

The literature suggests addressing the concern of limited program effect persistence through implementing the programs for longer periods of time [14, 29]. By doing this, higher program costs are unavoidable; hence, the benefit and costs of extending the program duration has to be evaluated by the program implementers.

### Limitations

Limitations of this study associated with external validity were present, as with most nutrition program evaluations. This study focused on a subset of the population which limits the generalizability of the results beyond this community. With respect to internal validity, one possible confounding issue is if there were any changes to federal or state level SNAP policy. There were no other store receipt data available to the authors, implying the assumption that these consumers only purchase their food from this supermarket. This assumption was not as restrictive as it may seem given that there were no other nearby supermarkets with similar assortment and quality. Finally, it should be noted that one limitation of expenditure analysis is that it does not capture changes in F&V choices and the differences in relative prices, which could be partially driving the expenditure changes.

## Additional files

Additional file 1: SNAP versus Non-SNAP Customers F&V Purchases Before DUFB. Study Supermarket Receipt Data. Receipt data from an independent supermarket in Detroit, Michigan that participated in the DUFB program was used for this analysis. The dataset includes all store transactions from May 2014 through January 2015. (DOCX 84 kb)

Additional file 2: Before versus During DUFB Implementation Regression Results. Study Supermarket Receipt Data. Receipt data from an independent supermarket in Detroit, Michigan that participated in the DUFB program was used for this analysis. The dataset includes all store transactions from May 2014 through January 2015. (DOCX 83 kb)

Additional file 3: During versus After DUFB Implementation Regression Results. Study Supermarket Receipt Data. Receipt data from an independent supermarket in Detroit, Michigan that participated in the DUFB program was used for this analysis. The dataset includes all store transactions from May 2014 through January 2015. (DOCX 79 kb)

Additional file 4: Before versus After DUFB Implementation Regression Results. Study Supermarket Receipt Data. Receipt data from an independent supermarket in Detroit, Michigan that participated in the DUFB program was used for this analysis. The dataset includes all store transactions from May 2014 through January 2015. (DOCX 75 kb)

Additional file 5: Summary of DUFB Effects over Time using Nonlinear Models. Study Supermarket Receipt Data. Receipt data from an independent supermarket in Detroit, Michigan that participated in the DUFB program was used for this analysis. The dataset includes all store transactions from May 2014 through January 2015. (DOCX 105 kb)

## Abbreviations

DD: Difference in Difference
DUFB: Double Up Food Bucks
F&V: Fruits and vegetables
FINI: Food Insecurity Nutrition Incentive
ITT: Intention to treat
SNAP: Supplemental Nutrition Assistance Program

## Footnotes

1 SNAP is a federal entitlement and nutrition program that provides money to low income Americans to purchase food at grocery stores, convenience stores, and some farmers’ markets and co-op food programs on an electronic benefits transfer card, which functions like a debit card.

2 The Kolmogorov-Smirnov equality of distributions test rejected the null hypothesis that the two group’s F&V expenditures have the same distribution. Hence, the analysis in this study is valid for identifiable customers but may not be representative of the entire store.

3 There were a total of 535 transactions that used a DUFB gift card as payment but the transactions which only used the DUFB gift card as payment do not have an identifier associated with them; hence could not be connected to an identifiable customer.
