# Supplementary material for: Double Up Food Bucks program effects on SNAP recipients' fruit and vegetable purchases
Source: BMC Public Health. 2017 Dec 12;17:946. doi: 10.1186/s12889-017-4942-z (PMC5727931; doi:10.1186/s12889-017-4942-z)
Supplement: Supplementary file 1 — SNAP versus Non-SNAP Customers F&V Purchases Before DUFB. Study Supermarket Receipt Data. Receipt data from an independent supermarket in Detroit, Michigan that participated in the DUFB program was used for this analysis. The dataset includes all store transactions from May 2014 through January 2015. (DOCX 84 kb) [file 12889_2017_4942_MOESM1_ESM.docx]

**Additional File 1: SNAP versus Non-SNAP Customers F&V Purchases Before DUFB**

The parallel trend assumption alleviates the endogeneity of the treatment concern for the DD estimators. It requires that in the absence of treatment, the average change in the F&V expenditure would have been the same for both the treatment and control groups. To assess this assumption the unconditional F&V expenditure patterns in SNAP versus non-SNAP before DUFB were compared. Figure A.1 shows the lines of best fit for SNAP and non-SNAP F&V purchases before the DUFB program. It shows that the SNAP F&V purchases over time were moving roughly together with the non-SNAP F&V purchases, which provides confidence that the parallel trend assumption was satisfied. Hence, the non-SNAP consumers’ transactions over time were used as the control group for analysis.
