# Supplementary material for: Double Up Food Bucks program effects on SNAP recipients' fruit and vegetable purchases
Source: BMC Public Health. 2017 Dec 12;17:946. doi: 10.1186/s12889-017-4942-z (PMC5727931; doi:10.1186/s12889-017-4942-z)
Supplement: Supplementary file 2 — Before versus During DUFB Implementation Regression Results. Study Supermarket Receipt Data. Receipt data from an independent supermarket in Detroit, Michigan that participated in the DUFB program was used for this analysis. The dataset includes all store transactions from May 2014 through January 2015. (DOCX 83 kb) [file 12889_2017_4942_MOESM2_ESM.docx]

**Additional File 2: Before versus During DUFB Implementation Regression Results**

|  | (1) | (2) | (3) | (4) | (5) | (6) |
| --- | --- | --- | --- | --- | --- | --- |
| Variables | **F&V**  **Exp** | **Fruit**  **Exp** | **Veg**  **Exp** | **F&V**  **Exp Share** | **F&V Variety** | **F&V Purchase Decision** |
|  |  |  |  |  |  |  |
| DUFB Effect | 0.404*** | 0.079 | 0.325*** | 0.007*** | 0.107** | 0.006 |
| SNAP | -0.344*** | -0.165** | -0.179** | -0.002 | 0.040 | 0.073*** |
| May 2014 | -0.490*** | 0.176** | -0.666*** | -0.006*** | -0.192*** | -0.018** |
| June 2014 | 0.322** | 0.656*** | -0.334*** | -0.003 | -0.140*** | -0.021*** |
| July 2014 | -0.011 | 0.540*** | -0.529*** | -0.010*** | -0.226*** | -0.040*** |
| August 2014 | -0.387*** | 0.471*** | -0.858*** | -0.011*** | -0.169*** | -0.036*** |
| September 2014 | -0.316*** | 0.294*** | -0.610*** | -0.010*** | -0.230*** | -0.045*** |
| October 2014 | -0.197* | 0.308*** | -0.505*** | -0.007*** | -0.208*** | -0.035*** |
| Other Dept Exp | 0.044*** | 0.018*** | 0.025*** | -0.000*** | 0.015*** | 0.001*** |
| Number of Visits | 0.488*** | 0.234*** | 0.254*** | 0.003*** | 0.191*** | 0.026*** |
| Constant | 0.405*** | -0.216*** | 0.621*** | 0.070*** | 0.588*** | 0.394*** |
|  |  |  |  |  |  |  |
| Observations | 43,600 | 43,600 | 43,600 | 43,600 | 43,600 | 43,600 |
| R-squared | 0.353 | 0.235 | 0.306 | 0.008 | 0.267 | 0.087 |
| Number of ID | 12,046 | 12,046 | 12,046 | 12,046 | 12,046 | 12,046 |

*** p<0.01, ** p<0.05, * p<0.1
