# Supplementary material for: Double Up Food Bucks program effects on SNAP recipients' fruit and vegetable purchases
Source: BMC Public Health. 2017 Dec 12;17:946. doi: 10.1186/s12889-017-4942-z (PMC5727931; doi:10.1186/s12889-017-4942-z)
Supplement: Supplementary file 4 — Before versus After DUFB Implementation Regression Results. Study Supermarket Receipt Data. Receipt data from an independent supermarket in Detroit, Michigan that participated in the DUFB program was used for this analysis. The dataset includes all store transactions from May 2014 through January 2015. (DOCX 75 kb) [file 12889_2017_4942_MOESM4_ESM.docx]

**Additional File 4: Before versus After DUFB Implementation Regression Results**

|  | (1) | (2) | (3) | (4) | (5) | (6) |
| --- | --- | --- | --- | --- | --- | --- |
| Variables | **F&V**  **Exp** | **Fruit**  **Exp** | **Veg**  **Exp** | **F&V**  **Exp Share** | **F&V Variety** | **F&V Purchase Decision** |
|  |  |  |  |  |  |  |
| DUFB After Effect | 0.068 | 0.059 | 0.009 | -0.001 | -0.077 | -0.015 |
| SNAP | -0.271* | -0.190** | -0.081 | -0.002 | 0.075 | 0.080*** |
| May 2014 | -0.464*** | -0.075 | -0.389** | -0.002 | -0.168*** | -0.004 |
| June 2014 | 0.430*** | 0.443*** | -0.014 | 0.005* | 0.049 | 0.015 |
| July 2014 | 0.073 | 0.309*** | -0.236** | 0.001 | -0.107* | -0.010 |
| December 2014 | -0.022 | -0.185*** | 0.163* | -0.001 | -0.125*** | -0.012 |
| Other Dept Exp | 0.043*** | 0.018*** | 0.025*** | -0.000*** | 0.015*** | 0.001*** |
| Number of Visits | 0.454*** | 0.217*** | 0.237*** | 0.003*** | 0.176*** | 0.025*** |
| Constant | 0.391*** | 0.118 | 0.274*** | 0.065*** | 0.569*** | 0.378*** |
|  |  |  |  |  |  |  |
| Observations | 31,121 | 31,121 | 31,121 | 31,121 | 31,121 | 31,121 |
| R-squared | 0.350 | 0.236 | 0.298 | 0.008 | 0.267 | 0.090 |
| Number of ID | 11,458 | 11,458 | 11,458 | 11,458 | 11,458 | 11,458 |

*** p<0.01, ** p<0.05, * p<0.1
