# Supplementary material for: Double Up Food Bucks program effects on SNAP recipients' fruit and vegetable purchases
Source: BMC Public Health. 2017 Dec 12;17:946. doi: 10.1186/s12889-017-4942-z (PMC5727931; doi:10.1186/s12889-017-4942-z)
Supplement: Supplementary file 5 — Summary of DUFB Effects over Time using Nonlinear Models. Study Supermarket Receipt Data. Receipt data from an independent supermarket in Detroit, Michigan that participated in the DUFB program was used for this analysis. The dataset includes all store transactions from May 2014 through January 2015. (DOCX 105 kb) [file 12889_2017_4942_MOESM5_ESM.docx]

**Additional File 5: Summary of DUFB Effects over Time using Nonlinear Models**

|  | Before  versus  During | During  versus  After | Before  versus  After |
| --- | --- | --- | --- |
|  | Average Change  (95% Confidence Interval) | Average Change  (95% Confidence Interval) | Average Change  (95% Confidence Interval) |
| F&V Expenditure  (Tobit) | $0.36***  ($0.12, $0.60) | -$0.29**  (-$0.57, $0.01) | $0.04  (-$0.25, $0.33) |
| Fruit Expenditure  (Tobit) | $0.08  (-$0.06, $0.22) | -$0.11  (-$0.27, $0.05) | -$0.01  (-$0.18, $0.16) |
| Veg Expenditure  (Tobit) | $0.27***  ($0.11, $0.43) | -$0.18*  (-$0.37, $0.00) | $0.05  (-$0.14, $0.24) |
| F&V Exp Share  (Tobit) | 0.70%***  (0.32%, 1.14%) | -0.60%***  (-1.01%, -0.21%) | 0.10%  (-0.41%, 0.60%) |
| F&V  Variety  (Poisson) | 0.04**  (0.010, 0.064) | -0.09***  (-0.12, -0.05) | -0.06  (-0.10, 0.02) |
| F&V Purchase Decision  (Probit) | 0.03  (-0.03, 0.08) | -0.03  (-0.09, 0.03) | -0.01  (-0.07, 0.06) |

*** p<0.01, ** p<0.05, * p<0.1

For the expenditure and expenditure share regressions Tobit models were estimated (due to a high prevalence of no F&V purchases in the dataset). For the F&V variety model a Poisson regression was estimated (because the variety variable is a count variable) and for the F&V purchase decision a probit regression was estimated (because the decision to purchase F&V is binary). Correlated Random Effects were estimated in these nonlinear regressions (time averages of the time varying customer specific explanatory variables included as additional explanatory variables) to avoid the incidental parameters problem present in fixed effects nonlinear models [24].
